# Efficacy of two antiseptic regimens on skin colonization of insertion sites for two different catheter types: a randomized, clinical trial

**DOI:** 10.1007/s15010-016-0899-6

**Published:** 2016-05-03

**Authors:** Juergen Thomas Lutz, Isabel Victoria Diener, Kerstin Freiberg, Robert Zillmann, Kija Shah-Hosseini, Harald Seifert, Bettina Berger-Schreck, Hilmar Wisplinghoff

**Affiliations:** 1 Department of Anaesthesiology, Intensive Care Medicine and Pain Therapy, St. Vinzenz-Hospital, Merheimer Str. 221-223, 50733 Cologne, Germany; 2 Institute of Medical Statistics, Informatics and Epidemiology, University Hospital of Cologne, Gleueler Str. 46, 50931 Cologne, Germany; 3 Institute for Medical Microbiology, Immunology and Hygiene, University of Cologne, Goldenfelsstr. 19-21, 50935 Cologne, Germany; 4 Laboratory Dr. Wisplinghoff, Classen-Kappelmann-Str. 24, 50931 Cologne, Germany

**Keywords:** Alcoholic disinfection, Catheter-related bloodstream infection, Octenidine dihydrochloride, Benzalkonium chloride, Central venous catheter, Epidural catheter, Bacterial recolonization

## Abstract

**Purpose:**

Catheter-related bloodstream infections affect patients in surgical and intensive care settings worldwide, causing complications, aggravation of existing symptoms and increased length of stay. The trial aimed at comparing two registered skin antiseptics with respect to their residual and therefore infection-preventing effects.

**Methods:**

In a parallel, monocentric, prospective, triple-blind, randomized trial the difference in bacterial recolonization of catheter skin sites in central venous (CVC) and epidural catheters (EC) was investigated by comparing two alcoholic-based skin disinfectants. Patients receiving planned surgeries or intensive care were eligible for the trial. Those in the trial group received skin disinfection with the additive octenidine dihydrochloride (OCT) (*n* = 51), those in the control group were treated with benzalkonium chloride as additive (BAC) (*n* = 59) prior to catheter insertion. Randomization was carried out by assigning patients to groups week-wise. Endpoints of the investigation were skin colonization of the catheter site counted in colony forming units per swab at three time points: (1) prior to catheter insertion, on untreated skin; (2) directly after catheter insertion, prior to sterile coverage; (3) 48 h after catheter insertion. The hypothesis was tested by a Wilcoxon test with a two-sided alpha = 5 %.

**Results:**

From second to third swab, recolonization of the catheter-surrounding skin was significantly lower in the trial group for both sorts of catheters: delta 2–3 OCT group: 0.72 (95 % CI: 0.42; 1.02); delta 2–3 BAC group: 1.97 (95 % CI: 1.45; 2.50); *p* < 0.001. None of the patients enrolled developed a catheter-related blood stream infection (CRBSI) during follow-up.

**Conclusions:**

Previous studies have shown that skin colonization is strongly associated with the occurrence of CRBSI. This randomized controlled trial supports the observations made in previous trials that octenidine dihydrochloride in disinfectants is more effective than agents containing other additives with regard to skin recolonization surrounding CVC and EC insertion sites. Therefore, it is likely to also reduce the risk of CRBSI in these patient groups. The trial was approved by the North Rhine Medical Association in July 2014 (application-no.: 2014222).

## Background

Central venous and epidural catheters are commonly needed in patients prior to surgery, a variety of invasive procedures or in an ICU setting [1, 2]. Many patients suffer from CRBSI originating from poorly disinfected or re-contaminated insertion sites around the catheter [3].

The minimization of catheter-surrounding skin recolonization is an effective step to avoid these infections [4], which are associated with many consecutive risks and cause an increase in treatment costs and lengths of stay [5]. As the amount of skin colonization is directly associated with catheter colonization and therefore with CRBSI risk, it is essential to focus on continuous skin hygiene [6].

Alcohol shows a strong immediate antimicrobial activity, but has no relevant residual activity on skin. Alcohol additives like benzalkonium chloride (BAC), chlorhexidine (CHX) or octenidine dihydrochloride (OCT) exert a different amount of residual activity on skin once the alcohol’s immediate effect has worn off [7]. OCT offers a broad and fast acting fungicidal, bactericidal and partly virucidal effect in a 0.1 % concentration [8].

Earlier trials have shown the residual activity of some of these disinfectants. Particularly, CHX as a strong residual agent shows clinical evidence to prevent catheter colonization and catheter-related blood stream infections (CRBSI) when using CHX/alcohol combinations prior to vascular catheter insertion and during catheter care [9].

Similar clinical investigations have proven a residual effect for octenidine dihydrochloride which leads to a decrease in skin recolonization after the alcohol has evaporated from the skin [10, 11].

The objective of this study was to compare an alcohol-based skin disinfectant containing OCT with regards to its residual effect to a reference alcohol-based skin disinfectant containing BAC.

## Methods

### Trial description

A randomized, prospective, controlled trial was conducted to compare the skin recolonization at central venous catheter (CVC) and epidural catheter (EC) insertion sites over 48 h in patients receiving a skin disinfection with either a BAC- or an OCT-containing alcohol-based disinfectant.

### Setting and participants

From July to December 2014, 216 patients in an academic teaching hospital in Cologne, Germany, were enrolled in the trial. The majority of patients received a catheter prior to surgery, all others (<2 %) were patients in the Intensive Care Unit (ICU).

The Ethical Review Board of the North Rhine Medical Association approved the trial in July 2014 (application-no.: 2014222).

Adult patients likely to receive a CVC or EC or both according to pre-operative standards were asked for their informed consent. Pre-trial exclusion applied for minors, emergency patients and patients with nosocomial infections. Patients whose swabs could not be obtained according to protocol were excluded. This applied for catheters being removed during the follow-up period as well as for patients whose sterile dressing did not cover the insertion site sufficiently after 48 h. Applying house standards according to National Hospital Hygiene Standards [12], all catheter insertions were carried out by experienced anesthesiologists, following a strict protocol including the use of surgical masks and caps, sterile gowns, gloves and catheter kits. The sterile dressing was applied right after the insertion, using a transparent foil to facilitate inspection of the insertion site. According to the study protocol, nurses or doctors changed the sterile cover every 48 h after disinfecting the catheter and its surrounding skin with a propranolol spray and a sterile pad.

### Randomization

The disinfection regimen was changed at weekly intervals, thereby randomly assigning patients to the trial or control group, respectively. The patients, the microbiological laboratory and the statistician were blinded to the assignment. The medical staff could not be blinded due to the colorant solely in the BAC agent and the use of transparent dressings.

After having given their informed consent, patients received skin disinfection with either one of the following commercially available and legally registered antiseptics:

#### Trial group

Propan-1-ol 30 % (w/w), propan-2-ol 45 % (w/w), octenidine dihydrochloride (OCT) 0.1 % (w/w) (octeniderm^®^, Schülke & Mayr, Norderstedt, Germany).

#### Control group

Propan-2-ol 63 % (w/w), benzalkoniumchloride (BAC) and coloring agents (Cutasept G^®^, Paul Hartmann AG, Heidenheim, Germany).

The dressing chosen for all patients was a sterile, transparent gauze designed for close skin site monitoring. The procedure for the control group was the established hospital standard, the trial group’s only alteration being the use of an alternative disinfectant. The staff only changed their routine throughout this trial by taking swabs at the fixed time points.

### Settings of the trial

All patients enrolled were treated and followed-up between July and December 2014 in the Department of Anaesthesiology at St. Vinzenz-Hospital, Cologne, Germany. Patients received catheters prior to surgery in the hospital’s departments of abdominal, thoracic, gynecological and traumatological surgery or as part of the intensive care or pain treatment in the ICU or the department of pain therapy respectively. The environment for catheter insertions was either the operating theater or the ICU.

Patients who were scheduled for catheters repeatedly during the same or various hospitalizations were allowed to be enrolled twice (respectively, three times) if at least 4 weeks had passed since the previous catheterization.

All adult patients scheduled to receive a CVC or EC who gave their written consent were included.

To reduce the loss of patients due to protocol deviations, the fixation especially for CVC was improved by applying strain-relief to the connected lines. In addition, all sterile covers were additionally fixated with adhesive tape on the rim. For EC patients, the sterile dressings’ size was increased and its rims supported by adhesive tape.

### Microbiological methods

All swabs were obtained by trained members of the trial team under standardized conditions using a sterile drape with a circular hole with a 7 cm diameter. The thereby marked skin surrounding the catheter insertion site was swiped with a sterile flocked swab (eSwab Liquid Amies, Copan, Brescia, Italy) with a movement ten times vertically and ten times horizontally. The tip was then immersed in the swabs' prefilled 1.0 mL sterile 0.01 M phosphate buffered saline (PBS) for transport to the microbiological laboratory.

To determine the bacterial burden, samples were cultured quantitatively (native, 1:10 and 1:100 dilution) on trypticase-soy-agar plates with sheep-blood (bioMérieux, Nürtingen, Germany). After vortex mixture, cultures were incubated for 36–48 h at 35 ± 1 °C. All tests were run in duplicate. The plates were inspected by a technician and the supervising microbiologist at 18–24 and 36–48 h. Results were expressed as CFU per swab.

### Outcomes and follow-up

Main outcome parameter was the difference in CFUs/swab (colony forming units per swab) of swab 3 (i.e. 48 h after insertion of CVC or EC) in comparison to swab 2 (i.e. immediately after disinfection and insertion). Secondary objective was the comparison of swab 3 and swab 1 (i.e. before disinfection and insertion). Patients were divided into cohorts primarily by skin disinfection regimen and secondly by type of catheter, so that four groups could be compared (CVC with OCT, CVC with BAC, EC with OCT, EC with BAC).

Patients’ comorbidity factors such as smoking, diabetes and permanent corticoid therapy were monitored. None of the enrolled patients suffered from chronic skin conditions. The amount of white blood cells and C-reactive protein were also taken into account.

Side effects, such as skin irritation (burning, itching, swelling, redness) were also closely monitored.

### Statistical analysis

Statistical analysis based on the CFUs counted by the laboratory personnel was statistically conducted by the University Hospital of Cologne’s Institute for Medical Statistics, Informatics and Epidemiology (Institut für medizinsche Statistik, Informatik und Epidemiologie, IMSIE).

The hypothesis was that octenidine as an additive to alcoholic skin disinfectants has a longer lasting antimicribiological effect on catheter-surrounding skin than benzalkonium chloride.

The analysis was conducted using the Statistical Package for the Social Sciences, version 22 (SPSS, IBM, Armonk, NY, USA). CFUs counted were logarithmized due to their great span. The disinfection regimen was compared at two time points: immediately after catheter insertion (swab 2) and 48 h after catheter insertion (swab 3). Swab 1 prior to skin disinfection was conducted as a baseline, as patients with different backgrounds were expected to start off with extremely different CFU amounts on their untreated skin. The hypothesis was tested by a Wilcoxon test with a two-sided alpha = 5 %.

## Results

216 patients were enrolled in the trial from July to December 2014. 106 patients had to be excluded from further analysis due to manipulation, partial or complete loss of the sterile dressing on the insertion site during the first 48 h. Other reasons for exclusion were disinfection within 48 h prior to first change of dressings or catheter-removal for therapeutic reasons, and the patient’s death or transferal to another hospital.

After having entered the trial, patients had to be excluded due to prematurely removed or loosened sterile covers on the catheter site. In addition, patients whose catheters had been removed in the 48 h follow-up period were excluded.

Patients entering the trial without any skin colonization in the first swab were excluded, as their skin swab result could not be considered a reliable marker. The last reason for exclusion was a decrease in skin colonization from swab 2 to swab 3.

The remaining 110 patients were divided into two main groups (trial, *n* = 51 and control, *n* = 59) and into four subgroups (trial CVC, *n* = 25; trial EC, *n* = 26; control CVC, *n* = 29; control EC, *n* = 30). A significant difference in delta swab 3 to swab 2 and in delta swab 3 to swab 1 was expected for both regimens.

### Group characteristics

Patients in the trial and control group, even when being catheterized for different reasons, showed similar characteristics regarding comorbidity, sex, age and physical constitution.

41 patients received both a CVC and an EC in one session, eight entered the trial twice, and one patient was enrolled three times in total. At least four weeks had to elapse after first catheterization to re-enter again.

### Skin colonization

**Fig. 1:**
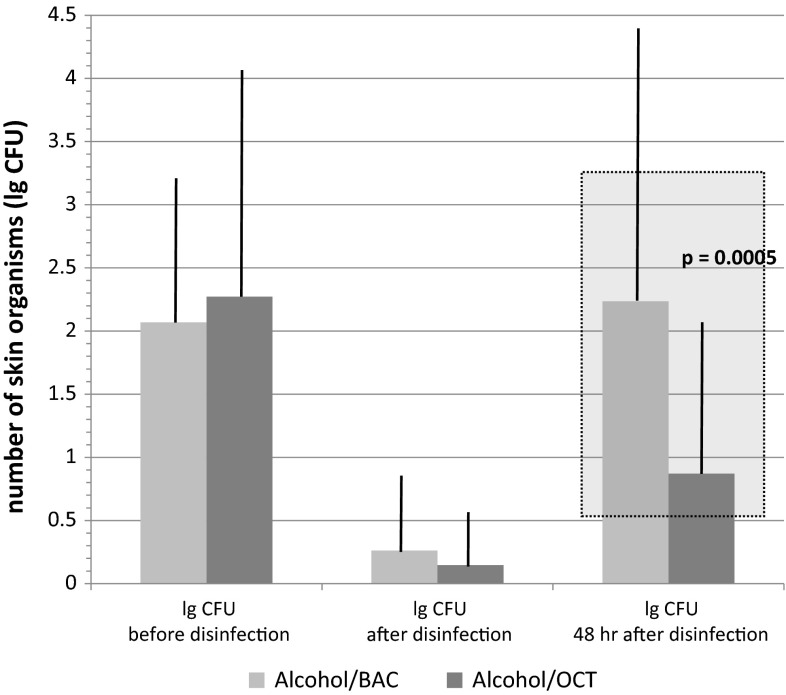
Number of organisms (CFUs/swab) at the insertion sites of catheters. CFUs/swab are displayed before, immediately after disinfection and 48 h after disinfection with Alcohol/BAC or Alcohol/OCT. The difference in colony counts between Alcohol/BAC and Alcohol/OCT after 48 h was statistically highly significant (*p* = 0.0005)

110 patients qualified for skin colonization analysis. There was no difference in the immediate effect (i.e. representing swab 2) between the trial and control group. The difference in CFUs from swab 2 to swab 3 showed a significantly lower value of recolonization in the trial group. In the subgroups, additionally divided by type of catheter, the values for CVCs were generally higher than for ECs in swabs 2 and 3 respectively, the level of recolonization again being much lower in the patients treated with OCT. Figure 1 shows the logarithmized amount of CFUs in all three swabs. The direct comparison of trial and control group shows the highly significant difference in recolonization from swab 2 to swab 3, as could be expected. The difference of mean values in patients treated with BAC was 1.97 (95 % CI: 1.45; 2.50), whereas the trial group treated with OCT only showed a delta of 0.72 (95 % CI: 0.42; 1.02; *p* = 0:0005).

### Side effects

**Fig. 2:**
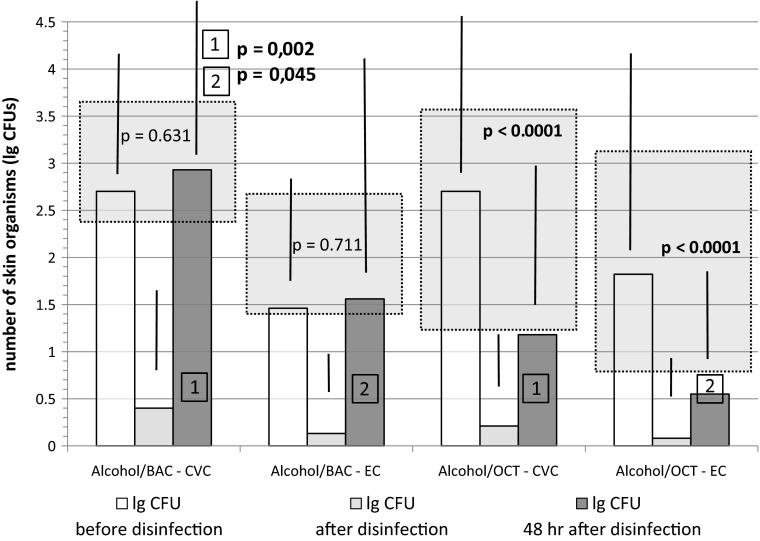
Colony forming units at the insertion sites of catheters. CFUs/swab are displayed before, immediately after and 48 h after disinfection with Alcohol/BAC or Alcohol/OCT. Sites for CVC and EC are shown separately. Statistical analyses were carried out within and between groups (1 and 2)

Side effects such as redness, swelling or pathological serum parameters were mild, rare and occurred in all groups. Few patients in all groups developed slight redness (*n* = 7, trial: *n* = 3; control: *n* = 4) or swelling (*n* = 3; trial: *n* = 2; control: *n* = 1) around the catheter insertion site (see Fig. 2; Table 1).

**Table 1:** Patient characteristics

	Alcohol/BAC		Alcohol/OCT	
	CVC	EC	CVC	EC
Number of patients	29	30	25	26
Catheter insertion sites	26/0/3	16/10/4	23/1/1	10/14/2
(CVC: jugular vein/subclavian vein/no specification)				
(EC: thoracical/lumbal/no specification)				
Age (mean)	67.4	66.47	66.8	66,69
Sex (f/m)	12/17	14/16	15/10	11/15
BMI (mean)	25.2	26.15	27	27.7
Diabetes (y/n/not specified)	7/18/4	6/21/3	6/16/3	9/16/1
ASA-classification (I/II/III/IV/ not specified)	0/2/13/1/3	1/15/12/1/1	0/10/11/3/1	0/12/14/0/0
Allergy of any type (y/n/not specified)	4/21/4	6/23/1	6/18/1	7/19/0
Nicotine abuse (y/n/not specified)	9/16/4	12/17/1	5/18/2	3/21/2
Corticoid therapy (y/n/not specified)	3/22/4	0/30/0	3/22/0	1/24/1
Max. CRP (mean)	19	12.15	14.42	9.3
Max. white blood cells (mean)	10.6	12	13.01	9.35
Redness after treatment	2	2	2	1
Swelling after treatment	0	1	1	1

### Complications

None of the patients developed signs of CRBSI or any other systemic side effects, which was expected due to the short period of follow-up.

## Discussion

This randomized controlled trial is the first to compare the immediate and mid-term efficacy over a 48 h period of two alcohol-based skin antiseptics used for disinfection of catheter insertion sites. The trial setup demonstrates similarities in the immediate effect and differences in the residual effect of the two alcohol-based skin disinfectants. The third skin swab taken 48 h after catheter insertion revealed a highly significant inhibition of regrowth of the skin flora in the Alcohol/OCT group in comparison to the Alcohol/BAC group. This residual effect of the disinfectant in the trial group can most likely be attributed to octenidine dihydrochloride, confirming results of other clinical studies with this formulation.

Skin colonization is a very important risk and surrogate factor for CRBSI [2, 6]. Therefore, the results suggest that the use of octenidine-based disinfectants may decrease CRBSI risk in CVC and EC patients significantly [6, 13].

For reasons of quality assurance the swab collection was limited to a small trial team whose members underwent a specific training. All swabs were conducted in the same manner, e.g. even applying a constant pressure while swabbing.

Limitations to the trial are the monocentric setup and the relatively small number of patients included in the statistical analysis. Nevertheless, regarding antimicrobial effect the regimens applied were highly different and resulted in clearly significant differences between groups. Furthermore, a long-term follow-up was not conducted, as the residual effect after initial disinfection was the focus of this study. Emergency patients, even though being at high risk for CRBSI [14], were excluded from the trial. For a long-term follow-up and thus a detection of potential differences in the incidence of CRBSIs, a much greater number of patients would have been needed to be examined, which was not possible in this monocentric setup. Furthermore, this study was not powered to answer this question.

The direct comparison of the current standard-antiseptic BAC and the octenidine-containing residual agent demonstrates the latter product’s performance and its potential clinical advantage.

The patients’ week-wise assignment to the groups prevented biases such as time of the day, temperatures in wards or physician in charge of catheter insertion. Generally, the patient population was homogenous as all of them met criteria for elective surgery that required catheterization in advance.

The high number of exclusions is a major limitation resulting from three phenomena: especially in the beginning of the trial, many sterile covers on patients’ insertion sites detached; the skin underneath could no longer be considered sterile. By standardizing the application of sterile dressings, the comparability of CFU results increased. Secondly, patients were excluded if they did not show any skin colonization in the first swab. Exclusion also resulted from a decrease in skin colonization from swab 2 to swab 3.

Furthermore, full barrier protection and close inspection for any early infection signs help to prevent CRBSI [5, 15, 16]. Regular training on hygiene measures and infection prevention also lowers the amount of CRBSI [17, 18].

## Conclusions

This RCT showed highly significant differences in microbiological skin colonization around CVC and ED insertion sites and therefore superiority of an alcohol-based skin disinfectant containing OCT in comparison to one containing BAC. As other independent trials have shown, OCT is a well-tolerated, highly effective skin disinfectant that might change the approach in catheter hygiene for both CVC [10, 11, 19] and EC.
